# Understanding the context of balanced scorecard implementation: a hospital-based case study in pakistan

**DOI:** 10.1186/1748-5908-6-31

**Published:** 2011-03-31

**Authors:** Fauziah Rabbani, Sabrina NH Lalji, Farhat Abbas, SM Wasim Jafri, Junaid A Razzak, Naheed Nabi, Firdous Jahan, Agha Ajmal, Max Petzold, Mats Brommels, Goran Tomson

**Affiliations:** 1 Department of Community Health Sciences, Aga Khan University, PO Box 3500, Stadium Road, Karachi, Pakistan; 2 Section of Urology, Department of Surgery, Aga Khan University, PO Box 3500, Stadium Road, Karachi, Pakistan; 3 Department of Medicine and Continuing Professional Education, Aga Khan University, PO Box 3500, Stadium Road, Karachi, Pakistan; 4 Department of Emergency Medicine, Aga Khan University, PO Box 3500, Stadium Road, Karachi, Pakistan; 5 Department of Family Medicine, Aga Khan University, PO Box 3500, Stadium Road, Karachi, Pakistan; 6 Nordic School of Public Health, PO Box 12133, SE-40242 Göteborg, Sweden; 7 Department of Public Health University of Helsinki, Finland and Medical Management Centre, Karolinska Institutet, Stockholm, Sweden; 8 Division of Global Health (IHCAR) Department of Public Health Sciences and Medical Management Centre, Karolinska Institutet, Sweden

## Abstract

**Background:**

As a response to a changing operating environment, healthcare administrators are implementing modern management tools in their organizations. The balanced scorecard (BSC) is considered a viable tool in high-income countries to improve hospital performance. The BSC has not been applied to hospital settings in low-income countries nor has the context for implementation been examined. This study explored contextual perspectives in relation to BSC implementation in a Pakistani hospital.

**Methods:**

Four clinical units of this hospital were involved in the BSC implementation based on their willingness to participate. Implementation included sensitization of units towards the BSC, developing specialty specific BSCs and reporting of performance based on the BSC during administrative meetings. Pettigrew and Whipp's context (why), process (how) and content (what) framework of strategic change was used to guide data collection and analysis. Data collection methods included quantitative tools (a validated culture assessment questionnaire) and qualitative approaches including key informant interviews and participant observation.

**Results:**

Method triangulation provided common and contrasting results between the four units. A participatory culture, supportive leadership, financial and non-financial incentives, the presentation of clear direction by integrating support for the BSC in policies, resources, and routine activities emerged as desirable attributes for BSC implementation. The two units that lagged behind were more involved in direct inpatient care and carried a considerable clinical workload. Role clarification and consensus about the purpose and benefits of the BSC were noted as key strategies for overcoming implementation challenges in two clinical units that were relatively ahead in BSC implementation. It was noted that, rather than seeking to replace existing information systems, initiatives such as the BSC could be readily adopted if they are built on existing infrastructures and data networks.

**Conclusion:**

Variable levels of the BSC implementation were observed in this study. Those intending to apply the BSC in other hospital settings need to ensure a participatory culture, clear institutional mandate, appropriate leadership support, proper reward and recognition system, and sensitization to BSC benefits.

## Background

As a response to the changing healthcare landscape, administrators in high-income countries (HICs) are implementing modern management tools such as the balanced scorecard (BSC) to improve hospital performance [1]. The BSC builds on the critical success factor (CSF) concept of a limited set of performance measures. It reports indicators in four different perspectives of equal weight: learning and growth, internal processes, customer satisfaction, and financial performance. Indicators can be developed from current data systems and used periodically for facilitating quality improvement and moving toward organizational excellence [2-4].

There is growing knowledge about the importance of organizational settings in implementing practices that are evidence-based [5,6]. One barrier that is continually identified towards implementation of successful performance measurement systems (such as the BSC) is the organizational context [7]. Contextual factors influencing efforts towards achieving goals include presence of a participatory culture, employee commitment and competence, technological resources, autonomy, degree of harmony between unit leader and employees, positive attitude towards the intervention being introduced, and supportive leadership [8,9].

Contextual analysis explains what works for whom under what circumstances [10]. This concept of realistic evaluation is based on the principle of generative causation -- *i.e*., what works is contingent upon the context (to whom and under what circumstances) in which initiatives are implemented. Acknowledgement of the need to incorporate the contextual setting is a new emphasis in BSC literature [4]. This is understandable because there are growing concerns about obstacles related to BSC implementation [11]. However, there is little guidance regarding which strategic processes are most effective under specific circumstances for successful BSC implementation. With the exception of Afghanistan [12], where the BSC was applied at the provincial level, the BSC has not to our knowledge been implemented specifically in hospital settings in low-income countries (LICs).

During 2005 and 2006, we designed and collected data pertaining to a series of studies on the BSC. These data were later analyzed, reported, and published. The first report [13] assessed the feasibility of using the BSC in the context of LICs and identified a team-oriented participatory (clan) organizational culture as a prerequisite for implementation. Subsequent studies [14,15] determined cultural readiness prior to BSC implementation and used group consensus methods to design a BSC for a tertiary care private hospital in Karachi, Pakistan.

Given the current knowledge gap between theory and practice, studies have recommended additional research focusing on contextual factors that facilitate or inhibit implementation of evidence-based practices [6]. In this regard, Pettigrew and Whipp's theoretical framework (PGF) of strategic change helps to understand the what, how, and why of the implementation process [6,16]. Building on the current science in implementation research, this study used aspects of the PGF model to explore contextual perspectives in relation to opportunities and challenges involved in BSC implementation in one hospital in Pakistan.

## Methods

### Setting and rationale for selecting study units

This study was conducted at a philanthropic, not-for-profit, private university hospital in Karachi. The hospital offers care to outpatients and inpatients of all socio-economic strata [17]. It has 542 beds in operation and offers a broad range of secondary and tertiary services to more than 38,000 hospitalized patients and approximately 500,000 outpatients annually. Its inpatients have an average length of stay of 3.9 days. The hospital has an International Organization for Standardization (ISO) certification and a Joint Commission International (JCI) accreditation [18].

The main clinical department in which this study was conducted has eight subspecialty sections with 54 full-time faculty members (49 male, 5 female), 67 residents (trainees, 40 male, 27 female) and 24 staff members. This department was also the focus of an earlier study on quantitative culture assessment [14]. Department refers to a large academic entity with responsibilities for teaching, clinical services, and research in a particular clinical discipline. It usually comprises various subspecialty sections that offer independent clinical services, though all sections administratively report to the department. A faculty is a trained person with relevant qualifications and experience commensurate with their academic rank. Major faculty assignments in clinical departments include teaching, research, and clinical services. All faculty members in this clinical department were physicians. Trainees are also qualified physicians completing postgraduate clinical training as part of a regular certified postgraduate medical curriculum. Staff refers to both doctors and allied health personnel in non-academic positions and includes mostly those of managerial rank.

This clinical department is part of the medical college. Faculty appointments for nurses fall under the domain of the school of nursing, a separate entity with its own goals for education and research. Nursing staff for patient care is appointed by the hospital nursing services. For quality improvement and patient care, doctors and nurses work together in the hospital but nurses do not have a direct reporting relationship to this department within the medical college. A director general of hospital services (highly qualified manager) and a medical director (senior physician) oversee all the hospital (medical, nursing, and allied) functions. Nurses were part of our sample during key informant (KI) interviews. In some participant meetings the concerned unit heads also invited their specialty-specific nurses to deliberate on the BSC indicators.

For the purpose of this study, Unit refers to the four clinical entities participating in BSC implementation. Out of the four, three were sections within this department while the fourth unit was a separate department (22 faculty, 6 staff) where pretesting related to the culture assessment tool was conducted previously [14]. These four units were selected (purposive sampling) based on the presence of a functional strategic plan, availability of baseline data on cultural typology and willingness to participate in the BSC implementation process.

These four study units will henceforth be referred to as Unit I to IV to maintain their anonymity.

### Study design

Because there is scant knowledge about the implementation of the BSC at the organizational level, we posed how/why-type questions to a real life situation. Case study was chosen as the preferred research method, as it is closely linked to the context in which it is being studied and is a research tool valuable for understanding dynamics present within a specific setting [19].

Case studies can be generalized against theoretical propositions that provide a blueprint to guide data collection. For this reason, theory development is an essential prerequisite prior to the collection of any case study data. Using PGF, our data collection approach was designed to provide examples of why (context), what (content), and how (process) of the BSC implementation process.

### Pettigrew and Whipp's theoretical framework

**Figure 1:**
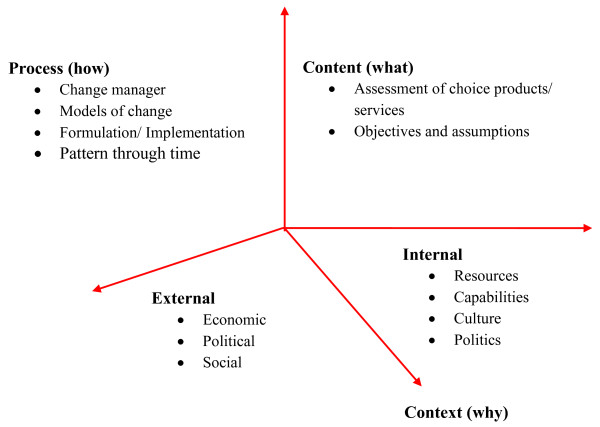
**The Dimensions of Strategic Change**. Source: Andrew Pettigrew, Richard Whipp. 1993. Managing Change for Competitive Success. Blackwell Publishing.

The PGF of strategic change has been widely used in analyzing and learning from change programs in organizations (Figure 1). Overall, the framework [16] focuses researchers and managers on three basic dimensions:

1. WHY of strategic change (with relevance to organizational context) encompasses elements of the healthcare environment in which BSC implementation takes place. Economic, political, and social factors at macro level constitute the external context. The internal context is characterized by organizational culture, leadership, human and financial resources, and type of healthcare setting.

2. WHAT of strategic change (influenced by internal context) is made up not only of overt, immediate, commercial, and financial objectives, but also implies the changes in key contextual elements during the process of BSC implementation.

3. HOW of strategic change denotes processes of organizational restructuring from strategy formulation through implementation.

Though these variables provide a language and common logic, the robustness of these variables is questionable, and no sharp distinction between process, content, and context can be drawn [20].

Aside from these essential dimensions of strategic management, certain related and seemingly useful central factors for managing such change are also described in the literature on PGF [16]. They are also referred to as factors for receptive contexts, and include environmental pressure, supportive organizational culture, change agenda and its locale, simplicity and clarity of goals, managerial clinical relations, key people leading change, and a policy's quality and coherence.

The magnitude of this study was small and its purpose was not to substantiate a theoretical model. Therefore, only the three basic PGF dimensions were considered to serve as a guiding lens for data collection and analysis. PGF emphasizes the continuous interplay and interaction between these change dimensions, which are assumed to act synergistically and collectively help to guide successful implementation [21].

### Research purpose and study questions

The research purpose was to study the implementation of the modern performance management tool, the BSC, in a private academic tertiary hospital in Karachi, Pakistan. The main operational study question was: 'What are the contextual circumstances under which the BSC is implemented in four study units of this hospital?'

Considering the importance given to context in PGF and the influence of context over process and content of implementation, PGF was chosen as the framework of inquiry and to pose secondary questions related to why, how, and what of BSC implementation (please refer to study results).

### BSC implementation

Prior to describing the actual BSC implementation, it is important to highlight the background and organizational context. The study hospital had an extensive health information system in place. In 2002, an internal situation analysis identified the need for better integration of data across various entities of the hospital for evidence-informed decision-making. It was recommended that academicians and administrators develop a road map together and foster a culture of teamwork, shared vision, and institutional ownership. The BSC was recognized as a road map for self-assessment and steady improvement towards excellence. In 2006, a multidisciplinary team composed of hospital leadership (medical director, chief operating officer on-site hospital services) agreed that the hospital could benefit from a BSC incorporating and integrating both clinical and non-clinical indicators. In 2008, a new vice president (VP) was appointed for hospital services, with past experience of serving as an executive director at Guy's and St. Thomas National Health Service (NHS) Foundation Trust in London. The newly appointed VP was responsible for corporate and clinical governance, clinical operations, and organization-wide performance measurement and management. Under the VP's direction, the BSC was envisaged as an organizational performance management pyramid empowering all levels (executive to operational) with varying metrics and details. It was anticipated that the BSC would serve to link the hospital's strategic plan with individual department objectives. The frontline level was to examine details with a large set of indicators tracked on a monthly/quarterly basis. It was concerned with problem solving and improvements, whereas the board and executive management would be more aligned toward long-term global trends, biannual summary reports and focused on overall strategy and governance. The first author was a part of these institutional deliberations and, based on this larger mandate and her own professional interest, undertook the task of conducting studies on the BSC as indicated above.

A generic BSC (hospital-level) with 20 indicators (Additional File 1) was developed. This had the core set of performance measures modified in each quadrant of the BSC based on the hospital's strategic priorities. However, for many employees, particularly in large organizations, the overall goals of the organization can seem too distant to be synergized with individual entity-level objectives [22]. Consequently, prior to introducing the BSC at an institutional level, the scorecard was tested at the frontline specialty (clinical department) level in this study. Three basic implementation steps were defined. First and foremost, the authors (first and second author primarily) were involved in sensitizing various subspecialties of the study department to the importance of the BSC via presentations in their specialty-specific monthly meetings. Four clinical units voluntarily opted to test the BSC approach. The second step was to facilitate development of customized scorecards for each of these four units (Additional File 1). In this regard, the authors facilitated restructuring existing management meetings around the scorecard and also assisted to schedule separate monthly scorecard meetings. The authors kept a participant observation diary to record interactions during these meetings. The third step in implementation was to encourage performance reporting from each of the four units using the BSC. Following a 12-month implementation, KI interviews were conducted in 2009 (by the first author in the presence of the second author, both trained in qualitative research methods) to determine employee perceptions on the contextual barriers and strategic processes involved in BSC implementation.

Following these three steps, subsequent BSC implementation was left to the discretion of the four implementation units. Our data collection methods ensured that this unit vibrancy and process of BSC evolution was appropriately captured. The comparable state of progress on implementation within each unit is described in the results section and Table 1.

**Table 1:** Comparative progress of BSC implementation in the four study units

	Unit I	Unit II	Unit III	Unit IV
**I. Sensitization to BSC and willingness to participate^a^**	yes	yes	yes	yes
**II. Developing a customized BSC^a^**	yes	yes	yes	no
**III. Reporting performance based on BSC^a^**	yes	yes	partially	no
**IV. Main motivating factors for implementing BSC^b^**	Non-financial incentives: co authorship, promotion, etc	Non-financial incentives: co-authorship, promotion etc, leadership communicating a clear agenda	Financial incentives in lieu of clinical time released	Financial incentives in lieu of clinical time released
**V. Barriers to BSC implementation^b^**	Lack of interest and role awareness, access to information	Lack of interest and role awareness, access to information	Lack of interest and role awareness, clinical work load, access to information, designated HR, hierarchical culture, derogatory leadership	Lack of interest and role awareness, clinical work load, access to information, designated HR, hierarchical culture, derogatory leadership
**VI. Strategies to implement BSC^c^**	Designated HR, specialty level ownership, incorporating in existing information system processes, regular unit meetings	Designated HR, specialty level ownership, incorporating in existing processes, regular unit meetings	Incorporating in existing processes, regular unit meetings	

Researchers used participant observation and interview notes to arrive at a consensus in order to compare progress in BSC implementation between the four units.

^a^Comparative unit progress is shown based on the three defined steps in BSC implementation.

^b^Comparative unit progress based on context, *i.e*., why do these units wish/not wish to implement the BSC.

^c^Comparative unit progress based on process, *i.e*., how do these units get BSC implemented and by using what strategies.

### Ethical considerations

Data collection for this study was approved by the institutional ethical review committee of the first author (vide ERC 464-CHS/ERC-05; ERC 1297-CHS/ERC-09). Neither the identity of individual participants nor the clinical units under consideration has been revealed.

### Data collection methods

This case study inquiry relied on multiple sources of evidence, the need for data to converge in a triangulation fashion, and PGF framework to guide data collection and analysis [19]. The three data collection techniques used in this study assisted in better understanding the contextual realities of the implementation process and are detailed below.

### Survey

The authors conducted a larger cultural assessment survey prior to BSC implementation [14]. A validated questionnaire [23,24] was used to obtain mean scores for culture typology based on the competing values framework (CVF). Based on underlying dimensions of flexibility/control and external versus internal orientation, the CVF (Additional File 2) articulates four basic cultural types [23]. Established on norms of affiliation, group (clan) culture emphasizes participatory decision-making, consensus building, ownership, and teamwork. The developmental (open) culture motivates risk-taking and innovation. In contrast, the hierarchical (bureaucratic) culture reflects the values and norms of bureaucracy ensuring formal rules and regulations. Finally, the rational/market culture assumes achievement through task completion and efficiency.

For the purpose of this study, we reanalyzed the data obtained previously at the departmental level [14] to comment specifically on prevailing culture type in these four participating units.

### Participant observation

Participant observation is considered an in-depth data collection technique that can be used within case studies for insightful understanding of contextual sensitivity [19,25]. This text is based on 40 meetings held in the four clinical units over a span of 12 months. A thorough documentation ensured that minutes were kept for all meetings held by all four units. It is to be noted that of these 40 meetings, some meetings (the monthly unit meetings) were large gatherings with more than 25 participants. Smaller specific meetings of the project working group with core staff from each unit were also conducted. The researchers explained their role clearly and honestly before each meeting. In the large staff meetings it was clarified by the head of the unit that the researchers were there to observe the interactions as the process of BSC implementation unfolded. In the smaller working group meetings, the researchers had a more proactive role in helping the unit staff design their customized scorecards. Non-verbal behaviors were also noted.

### Semi-structured interviews with KIs

Semi-structured interviews allow for a conversation to be developed around the area of interest and are excellent for documenting people's reasoning for their behavior and their understanding or misunderstanding of a particular issue or subject [26]. This was an important feature in our study. We explored stakeholders' own perceptions of how the BSC was being implemented using an interview guide (Additional File 3). The guide was developed using the PGF of strategic change and addressed the how, what, and why aspects of BSC implementation.

In 2009, semi-structured interviews of a selected sample of 12 KIs were conducted. Each interview lasted approximately 30 minutes. A written informed consent was obtained prior to each interview, and interviewees were assured that their personal identity would be kept confidential. Selection criteria for these KIs were that they should be knowledgeable about how the BSC was chosen, designed, and implemented. Our KIs included nine faculty from the implementing units (six men, three women), two senior female nurses and one departmental manager (male) who was present in most of the meetings. To ensure complete privacy, most interviews were conducted in the office of the interviewees by the principal investigator and the research intern. After conducting these 12 interviews, it was determined that no new information could be extracted about the strategic processes and contextual challenges of the BSC implementation process and that thematic saturation had been obtained.

The observational period for BSC implementation in this study was 12 months. The KIs at the time of the interview were still involved in BSC implementation. Therefore, their quotations referred to in the text are mostly in present tense.

### Data analysis

#### Quantitative data analysis

**Figure 2:**
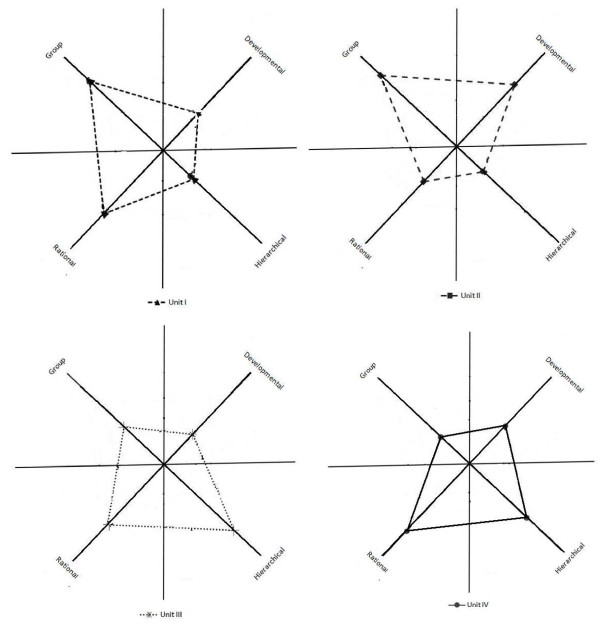
**Cultural profiling in the four BSC implementation units: quantitative survey**. Mean typology based on Competing Values Framework obtained through quantitative survey using validated questionnaire.

As part of the quality improvement implementation, respondents (faculty and residents) were required to indicate the extent to which their department/unit reflects characteristics associated with each culture type mentioned above. They were asked to 'share 100 points' between the four descriptions (copy of questionnaire available from the authors). Collating these point allocations provided a score (in the range 0 to 100) for each individual on the four cultural types. Data were analyzed at group level using standardized formulas for obtaining mean culture scores [24]. Obtained scores highlighted the context of prevailing culture type in the four clinical units (Figure 2).

#### Qualitative data analysis

This implied data abstraction emphasizing descriptions and interpretations based on the participant observations (meeting diary) and KI interviews (interview text). A summary sheet grouped main findings into common metrics. Like categories, metrics are defined to ensure that sufficient similar information is available to answer the questions posed by the study framework. This method of interpreting and analyzing information has been used elsewhere as well [27]. The same metrics can be used to answer several different questions because the information is cross-cutting. Some examples of metrics which helped to manage vast amount of information included: financial and non-financial incentives, role awareness, clinical workload, leadership support, human resources, data quality and access, culture, and BSC benefits. A simple storage and retrieval system was designed in QSR NVivo software 2.0 so that researchers could easily locate relevant information within metrics.

### Triangulation of methods

Triangulation is an authentic method verifying the repeatability of observations [28]. Reflections and reporting based on field notes from participant observation studies and other empirical data such as interviews are emphasized in ethnographic studies [25]. All sources of evidence in this study were reviewed and metrics were then mapped into the PGF framework in order to answer the why, what, and how questions related to BSC implementation (Table 2). Findings from the quantitative survey (Figure 2) were also consulted (method triangulation) to highlight the cultural context of BSC implementation. A schematic diagram of methodological triangulation is depicted in Additional File 4.

**Table 2:** Data triangulation based on Pettigrew's framework

*PGF Dimension*^a^	*Research Question*	*Corresponding metrics*^b^	*Selected Quotes* *(Key Informants) *^c^	*Observations* *(Meetings)*^d^	*Culture type* *(Survey)*^e^
**Context**	Why do these units wish/not wish to implement BSC?	Non-financial incentives	*Driving force should be there in the form of promotions, co-authorship etc (Units I, II)	*Units I and II were looking forward to non financial incentives to implement BSC	Unit I = Group and Rational
		Human resources	* We don't have anyone in the unit to be able to work on this (Unit IV)	*Unit III and IV more inclined towards financial incentives and attending to clinics	Unit II = Group and Development
		Clinical workload	* A hindering force in our unit is that people are overworked (Units II, IV)	*Lack of designated human resources, access to required information and time constraints were major barriers in Units III, IV	Unit III = Hierarchical and Rational
		Data quality and access	*We do not have ready access to all data (Units III, IV)		Unit IV = Rational and Hierarchical
		Benefits of BSC	* BSC reduces ad hoc reporting and improves outcomes (Unit II).	*Sensitization to BSC benefits facilitated implementation in Unit II	

**Process**	How do these units implement BSC?	Leadership, designated human resources, role awareness and ownership, regular meetings	*Our head has told us that BSC will give us the right opportunity (Unit II)	*Facilitatory factors were; role alignment and leadership communicating clear agenda for BSC (Units I and II)	Same as above
		BSC as part of ongoing information systems	* We are already using BSC but we don't call it so (Unit I)	* Introducing BSC as on ongoing information system activity/small scale (Units I and III)	
		Start small			

**Content**	What changes in key contextual elements occurred during implementation	BSC and culture	*What is required is a more participatory culture (Unit I).	Units I and II team-oriented Units III and IV; derogatory style of leadership	Same as above

^a^The key dimensions of Pettigrew's framework.

^b^Metrics are categories which accumulate similar data.

^c^Selected quotes from key informant interviews reflect the type of information contained in particular metrics.

^d^Participant observation from meetings correspond to metrics.

^e^Culture type of each unit was obtained through quantitative survey and highlights the background against which BSC implementation took place.

### Measures for achieving trustworthiness in the case study

Because a research design is to represent a logical set of statements, one can also judge the quality of a design using certain reasonable tests [19]. In this regard, several tests have been commonly used and are equally applicable and relevant to case studies. We have strived to use most of these tests to further elaborate the techniques used in our particular research (Additional File 5).

## Results

Information stemming from KI interviews, participation observations and the survey is triangulated (Additional File 4) and broadly described under the PGF dimensions of context, process and content. Most of the data clusters around context (why) -- the overarching dimension in PGF that influences process (how) and content (what). It is also important to clarify that the rich qualitative information obtained under each of the metrics was overlapping and cuts across more than one PGF dimension. Some examples of responses from the participants are quoted in italics.

### Context

The sub-question posed was: 'why do these four clinical units wish/not wish to implement the BSC?'

KIs mentioned that a main factor why they were involved in BSC implementation was anticipated organizational recognition in the form of financial (Unit III, IV) or non-financial incentives (Unit I, II). It was mentioned that a clinician's salary is not fixed and is dependent on the revenue generated through clinics and investigative procedures. Therefore, taking time out from patient care for BSC related work was very difficult. Moreover, there was pressure to maintain patient volumes by the hospital. It is to be noted (Figure 2) that the culture of Units III and IV was predominantly hierarchical (bureaucratic) and rational (goal-oriented).

'The problem is that the thinking is geared more towards financial incentives.' (Physician, Unit III)

'I think some driving force is needed for BSC implementation. This could be in the form of promotions, co-authorship, etc.; otherwise why would someone take interest?' (Physician, Unit I, Manager, Unit II)

It was also noticed (Units I, II, and III) during the participant observation that staff and faculty's prior experience of attending management workshops and involvement in hospital quality care initiatives was a strong reason why they positively considered BSC implementation.

'I am primed and sensitized to the whole concept of the scorecard. In 2006, I was involved in developing the quality improvement manuals. That is why I am interested in BSC implementation.' (Physician, Unit III)

Lack of political commitment and performance measurement initiatives at the national level (external context), combined with insufficient human resources to carry the BSC work forward were perceived barriers to why many staff and faculty thought BSC implementation was not a fruitful exercise. It is important to note that staff were both pre-committed with ongoing clinical work and also not inclined to contribute to an activity that would take time from their routine clinics and patient care.

'There is hardly any health system in Pakistan. The allocation to health is less than 1%. On paper everything appears to be organized, but the national picture is dismal. I am not sure therefore why we are doing this.' (Physician, Unit I)

'We do not have anyone in the unit to spare to be able to work on this. The manpower we have is overstretched in terms of clinical workload.' (Physician, Unit IV)

The above quotes from KIs were corroborated by participant observation. It was noticed during meetings of Units III and IV that the cell phones and beepers of clinicians were constantly buzzing and that participants were quickly distracted and left the room constantly. The researchers witnessed that during the meetings some faculty members were reading unrelated documents while others carried on mini-conversations amongst themselves.

Inaccessibility to required information remained a hindrance for monitoring BSC-related indicators.

All of the units mentioned that though it was easy to put the quality care indicators on the dashboard, patient satisfaction, employee satisfaction, and financial information by each specialty were difficult to obtain. These measurement issues with existing indicators have already been described in our earlier study [15].

'We are still struggling with the fact that information generated centrally should flow back to the peripheral department/unit and that is why we have not had a meeting on BSC last month.' (Physician, Unit I)

'Getting information about employee satisfaction in our unit is the weakest link.' (Physician, Unit II)

Moreover, in a Unit I meeting, it was observed that the designated employee contacted the source entity for obtaining information on aspects of patient satisfaction in his unit. But he could not obtain the required information.

Though Units I and II had some skepticism about the BSC, these units were relatively more positively geared towards the benefits of the BSC as compared to Units III and IV and hence made better progress towards implementation.

'BSC will provide a way to communicate efficiently. Right now information sharing and discussion only takes place on an *ad hoc *basis -- *i.e*. if something goes wrong. That is why I and my unit are very interested to participate.' (Physician, Unit II)

'Before, we had to strive to understand performance indicators but with BSC we can document when we have achieved our targets and that is why we are interested to move ahead.' (Manager, Unit I)

Unit III was quite satisfied with only reporting performance on its quality care indicators, and did not seem to comprehend how BSC would add value. Therefore, they lagged behind initially.

'I think most of the people are trying to understand the BSC but you know it is a new thing for us and the staff is not very clear about its purpose and importance.' (Physician, Unit III)

'One reason why people are not genuinely interested to take this forward ... they can't see the improvements in patient satisfaction, meeting clinical targets, etc.' (Physician, Unit III)

Another important inspiration for BSC implementation for Units I and II was the presence of conducive unit leadership and a cohesive team (participatory culture). Because heads of Units III and IV (more elements of hierarchical culture) were not personally motivated to take this work forward, these units lagged behind.

'This is not the right time for us to be involved when we are undergoing our own internal reorganization. Perhaps someone else should come and do it for our unit.' (Physician, Unit IV)

'Even if I want a pillow for my patient, it is not provided in time, then how can I assume that a task as complex as BSC can be accomplished by our unit.' (Physician, Unit III)

Units III and IV leadership could not clearly communicate the organizational agenda for BSC implementation, and hence it could be seen during meetings that employees confused the BSC with another top-down quality care initiative. Some also said they felt that the BSC's holistic approach took away their power/threatened their job, while others believed it to be a mere research project in which the first author had a vested interest.

'I don't want to push them for implementation of your project -- unless they themselves say that they would like to work on it.' (Physician, Unit III)

'If the clinicians start doing this type of work, my job will be at stake.' (Manager Unit III)

'Yes we can work on BSC but not now because our quality improvement report requires completion first.' (Physician, Unit IV)

### Process

The secondary study question was 'How do these four clinical units get BSC implemented and by using what strategies?'

Several strategies emerged: leadership appointing designated human resources, defining the role of staff and faculty in BSC implementation, developing a clear communication strategy, and promoting employee ownership of the process. Units I and II had assigned clear roles to their faculty and staff to shortlist indicators for the BSC and keep it in the agenda of their regular meetings. This is how reporting related to BSC was initiated in their meetings within the first six months of implementation. For Unit III, where ambiguities existed about clinicians monitoring indicators, few workshops and special meetings helped to clarify the concepts.

'Staff should clearly know their role in BSC implementation and how it will affect them.' (Physician, Unit I)

'Information about BSC benefits should trickle down to the lowest staff level with a sense of ownership.' (Nurse, Unit II)

'Our head has told us that BSC will just give us the right opportunity to make the difference.' (Physician, Unit II)

'The ownership should not be put on management only but on all the people doing the work.' (Physician, Unit I)

'We have to take the control in our hands which begins with selecting and monitoring our specialty level indicators.' (Physician, Unit II)

Another interesting strategy reported during the interviews was the inclination to incorporate the BSC into ongoing information system processes rather than introducing it as an entirely new initiative. Units were less skeptical if they were told that they could start a BSC with minimal indicators. Unit III was ready to begin with just two quadrants of the BSC until information on other desirable indicators was readily available.

'What we need to do is to reinforce that the BSC is already in place and we are just formalizing it.' (Physician, Unit I)

'We are already monitoring quality indicators which could be one quadrant of BSC...it's just that we don't call it so.' (Nurse Manager, Unit IV)

Emergent signs of change in the unit's culture could be noticed when one or more of these strategies were applied. They are referred to in the following Content section. A cross-case comparison of these units in terms of context and process is illustrated in Table 1.

## Content

In this PGF dimension, the sub-question being asked is: 'What changes occur in key contextual elements while implementing BSC?' It is noteworthy that culture is a key contextual element in PGF. Organizational culture is an emergent property, and cultural transformation is a complex multi-level and uncertain process that unfolds over many years [9]. In this section, the baseline culture of the implementing units is described (Figure 2) and an effort is made to capture early signs of emergent change in a unit's team dynamics while implementing the BSC.

The heads of Units I and II ensured that designated human resources were assigned for moving BSC implementation forward.

'I have already mentioned that the BSC is in line with the policies of our unit and I have designated two staff to work with you.' (Physician, Unit I)

Participant observation of Units I and II demonstrated that the atmosphere was relaxed and congenial. Faculty and staff sat in a classroom-style of setting, with the head of the unit seated amongst them. Despite initial reluctance among participants, a change was noticed after two to three meetings. Designated staff independently started presenting progress against the selected indicators in each of the four BSC quadrants.

In Unit III (predominantly hierarchical culture), it was noted that head of the unit was seated separately at the executive seat of the table during meetings. He attempted to answer all questions himself.

'Our culture currently is very individualistic; *i.e*. people feel that they don't have much say in decision making. What is required? We need to diffuse this and promote a team-oriented culture.' (Physician, Unit III).

It can therefore be assumed that a perceived need for change was present. Change started appearing slowly once BSC was regularly added to meeting agendas. Most of the staff and faculty progressively took ownership, as evident through their involvement in discussing and reporting the BSC indicators as part of their existing QMIS (Quality Management Information System).

In Unit IV, the long chain of bureaucracy delayed decision-making at each step of BSC implementation.

'I have all of the information required for BSC but what I need is approval from my nurse supervisor.' (Staff nurse, Unit IV)

In this type of relatively constrained atmosphere, none of the strategies discussed in the Process section were useful, no contextual change began and BSC implementation could not materialize.

## Discussion

To our knowledge, this is the first hospital-based case study describing BSC implementation in a LIC setting. It provided a unique opportunity for managers and physicians to explore their contextual perspectives in relation to opportunities and challenges involved in BSC implementation.

PGF theoretical construct served as a sufficient blueprint for data collection and analysis. Information from survey, semi-structured KI interviews, and participant observations were triangulated and mapped onto the three dimensions of PGF (Table 2). This mode of'analytic generalization,' utilizes a previously developed theory to compare the empirical results of the case study [19]. Other studies have also used PGF to understand implementation of a change process [6,29,30]. Syntheses of findings from similar multi-method studies have been reported in the literature of organizational studies [31,32].

The importance of organizational support (context) with regard to financial and non-financial incentives and prior work experience on quality care initiatives were highlighted as potential facilitating factors for BSC implementation. Such organizational support has also emerged as a critical factor in other studies [33]. Units I and II (predominantly participatory culture as assessed through the quantitative survey) considered non-financial incentives to be equally strong motivators for implementing the BSC. In contrast, Units III and IV (predominant culture type: bureaucratic and goal-oriented) strongly linked BSC implementation to financial gains, and it was observed and quoted during interviews that taking time out of clinical activity and investing in BSC implementation was a potential financial loss and distraction from pre-conceived goals. Similar context with emphasis on generating revenue has also been noted in other hospital-based studies [34].

BSC contextual barriers that surfaced in all units included clinical workload, lack of national performance management initiatives to provide benchmarks for comparison, an inability of leadership to communicate a clear BSC agenda, a lack of designated human resources, and ill-defined staff roles in BSC implementation. Paucity of comparable indicators from peer health units in the four BSC quadrants has also been reported from a recent study in Ontario's public health units [35]. Moreover, role awareness has also been cited as an important method of avoiding territorial conflicts in other settings [36]. Similar challenges in BSC implementation have been discovered in healthcare provider organizations in the United States. They include acceptance towards implementation, maintaining simplicity, and staff commitment [37]. During the interviews in Units III and IV, it was clear that there was difficulty in tracking BSC indicators because data were not readily available and accessible in the required formats. These results resonate with the findings of a nested qualitative study in England [38]. Deficiency of good quality data, unclear program direction, and a low level of awareness have also been identified as implementation barriers in a case study of nursing in Canada [39]. Due to these issues with data acquisition, Unit III, for instance, decided to bank on existing clinical quality indicators to initiate BSC implementation. Another study also concluded that the BSC could build on existing frameworks [40]. A multi-method study of organizations in Norway has come up with similar recommendations to start small [31].

Additionally, staff and faculty in our study perceived that BSC indicators such as employee and patient satisfaction were non-clinical in nature and therefore not of direct concern. They perceived that it was the role of hospital managers to keep track of the information, while the clinician's role was to concentrate on direct patient care. Such barriers between professional domains have also been noted in France [32]. Axelsson describes territorial barriers between professionals and administrators to be a classic concern within organizations [36]. Furthermore, during staff meetings of Units III and IV, it was observed that beepers and cell phones were a constant source of distraction. Clinicians in these two units seemed more interested in attending to calls from their clinics as opposed to focusing on BSC reporting. This context explains the competing priorities due to which these two units lagged behind in implementation compared to Units I and II.

Another contextual observation was that if employees were appropriately sensitized to the BSC benefits, it translated into a positive impact on implementation. Because Units I and II seemed quickly able to grasp the advantages of the BSC, implementation began sooner. Unit III had a delayed start, as participants initially failed to understand the added value of the BSC. Moreover, the designated employees in Unit IV had anxieties and fears that this new requirement of BSC-based performance reporting would be very time consuming. Unit IV therefore remained in a preparatory phase without entering actual implementation. A similar lack of understanding about the benefits of the BSC has been observed in Germany [41].

Strategies found useful in setting up a process of change and facilitating BSC implementation (process) included: providing designated human resources for monitoring of BSC indicators; ownership from all employees; communicating a clear agenda to implement the BSC; encouraging non-financial incentives; and reporting of BSC indicators in routine unit meetings. Other studies have also reported the importance of having open channels of communication within a workforce [42]. Introducing the BSC as an ongoing activity was found to be an important strategy, which was particularly effective in Unit III. Such a use for the BSC has been described in a study in the United States, in which the BSC was noted as an 'integrated information system' [43]. Unit II did not pose any objections to viewing the scorecard as a new initiative. Both Units I and II had two personnel each assigned for working on the BSC throughout the observed 12-month implementation period, and there was less emphasis on generating revenue. It is noteworthy that despite the perceived need for change to improve standards of care, Unit IV lagged behind as it encountered most of the implementation barriers and was unable to successfully employ any of the above strategies.

It is also essential to mention that Units I and II handle a large patient load on an outpatient basis and provide non-invasive diagnostic and therapeutic services. Units III and IV provide outpatient and inpatient services and are responsible for more invasive investigations with an emphasis on revenue generation and the maintenance of clinical volumes. This is a potential explanation why Units I and II adapted more readily to BSC implementation. Recent studies in Italy have also concluded that introducing the BSC to improve management of day-care surgery and gastroenterology endoscopy units has the potential to optimize services [44,45].

The BSC performance was closely linked with the prevalent culture (internal context) and the changes brought about in a unit's climate (content) as part of the implementation process. It has been mentioned that context greatly influences the how and what dimensions of PGF, and it is difficult to demarcate boundaries between the three dimensions. Culture is seen as a common base for values and understanding of principles within a professional organization [29]. Other studies on improving hospital performance have recognized the importance of human relation dimensions [32]. The culture types in the quantitative survey (Figure 2) matched the KIs opinion regarding the unit's culture and concomitantly what was observed during meetings of the unit. The purpose of this study was not to bring about a cultural transformation, but rather to understand how existing unit culture influenced the implementation process and what changes (if any) emerged in a unit's dynamics while implementing the BSC. It was noted that in Units I and II, which predominantly had participatory cultures, BSC implementation was enhanced. By contrast, in Unit III (bureaucratic culture) and Unit IV (goal-oriented culture), BSC implementation lagged behind. Unit III's relatively bureaucratic style prevented an early BSC implementation; the leadership of the unit appeared interested, but seemed very disparaging in assigning tasks to their staff and faculty. The culture of the unit gradually started showing signs of teamwork once the BSC continued to appear on the agenda of their regular meetings. Towards the end of the implementation period, the designated employees of Unit III took on the responsibility and ownership for BSC-based performance reporting in their monthly unit meetings. The importance of having such frequent formal and informal meetings with employees and managers is a sign of a participatory culture and has been shown to bring about support for improvement efforts and implementation initiatives [46]. In Unit IV, there was neither a fixed schedule nor a proper agenda for meetings; this lack of cohesive management and resistance to change impaired BSC implementation.

It is noteworthy to mention here that the assessment of culture typology is based on cross-sectional survey conducted earlier. At the outset, stakeholders were informed that this survey would highlight their readiness for quality improvement implementation based on the contextual information they provided about their unit. The same survey was used to understand the cultural typology of the four study units in which BSC implementation later began. Although some emerging signs of change were noted, no cause-and-effect relationship between the BSC and organizational culture is implied in either direction. During observations and interviews, stakeholders knew that the process of BSC implementation was being studied without a specific reference to the role of culture in the implementation process.

## Limitations

This study was not without limitations. It is based in just one private academic hospital in Pakistan and therefore findings are mostly relevant to this case. At least five other private tertiary hospitals in the country are comparable to the study hospital in terms of skilled manpower, diagnostic and curative facilities, and information technology infrastructure. Nevertheless, the study hospital is distinctive in the LIC setting because of its state-of-the-art facilities and international accreditation and certifications. Applicability of our findings to an audience outside non-academic settings should therefore be carefully interpreted. Due to logistic reasons and a short observation period, hardcore BSC outcomes (improvement of clinical indicators, patient and employee satisfaction, *etcl*.) could not be assessed. Similar shortcomings have also been noted in a recent study of BSC implementation in three acute care hospitals in a HIC setting [41]. Despite these limitations, the involvement of four hospital units in the BSC application and the study of the context of implementation was a unique experience with catalytic validity. The latter implies that our results are not merely descriptive, but part of a continuous process of change and, based on current experience, have the potential to guide future BSC implementation efforts. Such approaches have been considered very helpful in understanding how and why certain activities produce certain effects during an observational follow-up period [47].

The strategies used to increase trustworthiness of the findings (Additional File 5) in this study included theory-guided data collection and analysis. It is important to note, however, that the author of this article and the study subjects worked for the same organization. Given the lack of expertise in the field, in some participant observation meetings the researchers were also partial facilitators; this has the potential of introducing an observation bias and affecting team dynamics. It is possible that Units I and II showed greater enthusiasm because of the presence of the researchers in their meetings (Hawthorne effect). Much influence of the Hawthorne effect however, seems unlikely because Units III and IV consistently lagged behind despite facilitation by the researchers. Later, these observations were corroborated by interviews to increase the objectivity and neutrality of results.

For the purpose of this case study, the three PGF dimensions were used to guide the contextual and process analysis, and to look for patterns, identifying gaps in the BSC implementation. The purpose was not to confirm or refute the PGF theoretical model. Therefore, findings have not been described under the umbrella of the classical PGF factors. Future research tracking contexts over a longer period of time could examine the impact of the entire PGF or alternative strategic change frameworks across a variety of organizations with theoretical explanation building.

## Conclusions

A participatory culture, supportive leadership, financial/non-financial incentives, and support for the BSC in policies, resources, and routine activities appeared as desirable attributes. Role clarification and consensus about the purpose and benefits of the BSC were noted as key strategies for overcoming barriers related to BSC implementation. Similar drivers and blockers of performance management implementation have been reported from a synthesis of five case studies in the United Kingdom [48]. Moreover, it was realized that rather than seeking to replace existing information systems, initiatives such as the BSC could be readily adopted if they are built on existing infrastructures and data networks. Other studies have also pointed out the need to foster BSC champions, not rushing the BSC's introduction, creating a receptive organizational culture and integrating the scorecard with existing management processes [49].

## Competing interests

The authors declare that they have no competing interests.

## Authors' contributions

FR designed, planned, executed, analyzed, and wrote all drafts of the manuscript. SL assisted in conducting the interviews, data transcription, qualitative analysis and worked on several revisions of the manuscript. FA and WJ guided the larger institutional mandate on taking the BSC-related work forward and in reviewing draft manuscripts critically. JR, NN and FJ facilitated BSC implementation process and commented on draft manuscript. AA assisted in thematic content analysis of the qualitative data and MP facilitated the quantitative analysis and triangulation aspects of data. MB and GT critically reviewed the methodology, design, concept and data from the study and gave detailed feedback on several draft manuscripts.

All authors have read and approved the final manuscript.

## Supplementary Material

Additional file 1 **Institutional level scorecard developed in earlier study and customized BSCs for each of the respective units as part of the BSC implementation process in the current study presented in a tabular form**. Click here for file

Additional file 2 **Organizational culture competing values model used to illustrate the culture of the 4 study units**. Click here for file

Additional file 3 **Key informant interview guide developed based on PGF model**. Click here for file

Additional file 4 **Diagrammatic representation of methodological triangulation in this case study**. Click here for file

Additional file 5 **Research tactics used to strengthen this case study presented in a tabular form**. Click here for file
