# Supplementary material for: Understanding the context of balanced scorecard implementation: a hospital-based case study in pakistan
Source: Implement Sci. 2011 Mar 31;6:31. doi: 10.1186/1748-5908-6-31 (PMC3080822; doi:10.1186/1748-5908-6-31)
Supplement: Additional file 1 — Institutional level scorecard developed in earlier study and customized BSCs for each of the respective units as part of the BSC implementation process in the current study presented in a tabular form. [file 1748-5908-6-31-S1.DOC]

**Additional file 1: BSC indicators customized by the four clinical units**

| **BSC Perspectives** | **Hospital BSC** | **Unit I:** | **Unit II:** | **Unit III:** | **Unit IV:** |
| --- | --- | --- | --- | --- | --- |
| **Financial Perspective** | *Average charges  *Length of stay  *Daily census  *Net operating margin | *Average charges  *Daily census  *# of referrals from outpatient low cost clinic | *Average charges  *Daily census  *# of referrals to inpatient  * positive or negative variance | *Average charges  *Length of stay  *Daily census  *Net operating margin |  |
| **Internal Business Perspective** | *Laboratory report turnaround time  *Radiology film reject rate  *Unplanned stay after daycare procedure  *Incidence of .blood transfusion reactions  *Nosocomial infection ratte  *Cross match to transfusion ratio  *Needle stick injuries | *Appropriate  patient management  *Appropriate # of medicines per diagnosis  *Student’s satisfaction with teaching  *Satisfaction knowledge & skills of trainees  *# of adverse events  *Compliance with safety procedures and infection control  *Publication & research output | *Return ER visits within 48 hours  *Laboratory report turnaround time within 2 hours  * Patients admitted from ER within 4.5 hours  *Patients sent home within 2 hours of registration  *# of patients left against medical advice | * Length of hospital stay in cirrhotic patients with spontaneous bacterial peritonitis  *Length of stay in patients with upper G.I bleed  *Timely discharge of G.I patients from the wards  *Compliance with clinical guidelines (consultant credentials, conscious sedation and privacy maintained)  * Inpatient mortality rate |  |
| **Human Resource Perspective** | *Satisfaction with job  *Satisfaction with colleagues  *Satisfaction with on campus facilities  *Satisfaction with organization  *Satisfaction with supervisors | **,,** | **,,** | **,,** |  |
| **Patient Satisfaction Perspective** | *****Satisf action with physician  ***** Patient complaints  *Satisfaction with nursing  * Proportion of patients recommending hospital to others | **,,** | **,,** | **,,** |  |

The unit specific scorecards were customized based on routinely available hospital data. The institutional level scorecard was developed by the authors in earlier study .
