# Supplementary material for: Understanding the context of balanced scorecard implementation: a hospital-based case study in pakistan
Source: Implement Sci. 2011 Mar 31;6:31. doi: 10.1186/1748-5908-6-31 (PMC3080822; doi:10.1186/1748-5908-6-31)
Supplement: Additional file 2 — Organizational culture competing values model used to illustrate the culture of the 4 study units. [file 1748-5908-6-31-S2.DOC]

**Additional file 2: Organizational culture: competing values model**


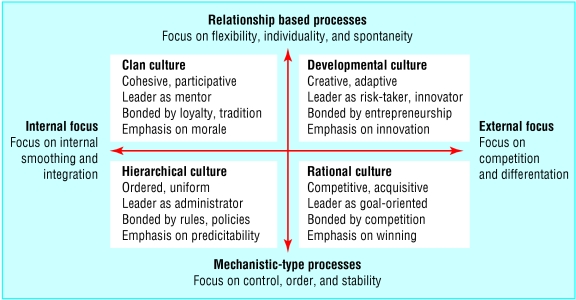


Source: Zammuto RF, Krakower JY: **Quantitative and qualitative studies of organizational culture.** *Research in Organizational Change and Development* 1991, **5:**83-114.
