# Supplementary material for: Understanding the context of balanced scorecard implementation: a hospital-based case study in pakistan
Source: Implement Sci. 2011 Mar 31;6:31. doi: 10.1186/1748-5908-6-31 (PMC3080822; doi:10.1186/1748-5908-6-31)
Supplement: Additional file 3 — Key informant interview guide developed based on PGF model. [file 1748-5908-6-31-S3.DOC]

**Additional file 3: Key informant interview guide**

| What was the main incentive to start/continue BSC implementation in your unit?  Has the implementation of BSC been guided by any performance measurement initiative at national level?  Has the implementation of BSC been guided by any performance measurement initiative at the hospital /organizational level?  Have you customized the BSC to your unit. If so how?  How was the BSC different from what performance measurement systems already existed in your clinical unit?  Exactly which activities were planned to implement the BSC?  Did your unit receive proper training in aspects of BSC implementation?  Were appropriate resources allocated for BSC implementation  Has the implementation of BSC affected the culture in your unit? If so how can you describe the shift  What were the effects of introducing BSC so far for your unit’s staff and faculty  Was your unit’s leadership conducive to BSC implementation? In what ways?  What helped and hindered the BSC implementation activities in your unit and what could have been done differently?  Did you have problems in accessing information to design and monitor BSC indicators?  Has BSC become part of your unit’s performance measurement system? |
| --- |

Interview guide developed based on Pettigrew and Whip’ s conceptual framework (WHAT, WHY, HOW)

Andrew Pettigrew, Richard Whipp. 1993. Managing Change for Competitive Success. Blackwell Publishing
