# Supplementary material for: Understanding the context of balanced scorecard implementation: a hospital-based case study in pakistan
Source: Implement Sci. 2011 Mar 31;6:31. doi: 10.1186/1748-5908-6-31 (PMC3080822; doi:10.1186/1748-5908-6-31)
Supplement: Additional file 4 — Diagrammatic representation of methodological triangulation in this case study. [file 1748-5908-6-31-S4.DOC]

**Additional file 4: Diagrammatic presentation of methodological triangulation**

Qualitative

Participant observation

Key informant interviews:

Culture survey

Valid. q

4 units

Context of BSC implementation described: WHAT, WHY , HOW

Chain of Evidence

Quantitative contex

**RESEARCH OBJECTIVE**

Describe the context of BSC implementation: WHY, WHAT, HOW

Multiple Sources of Evidence

Data Bases

Data Triangulation: Converging lines of inquiry based on PGF frameworkheoreticalmodel
