# Supplementary material for: Understanding the context of balanced scorecard implementation: a hospital-based case study in pakistan
Source: Implement Sci. 2011 Mar 31;6:31. doi: 10.1186/1748-5908-6-31 (PMC3080822; doi:10.1186/1748-5908-6-31)
Supplement: Additional file 5 — Research tactics used to strengthen this case study presented in a tabular form. [file 1748-5908-6-31-S5.DOC]

**Additional file 5: BSC implementation case study**: research tactics used to strengthen the design

| **Tests** | **Case Study Tactic** | **Phase of research in which tactic occurs** |
| --- | --- | --- |
| **Construct Validity** | *Using multiple sources of evidence to give a rich empirical account of the BSC implementation: participant observations, semi-structured key informant interviews and surveys were triangulated  *Having key informants review interview draft notes and make comments  * Similar grounds on context of BSC implementation were covered in all 3 data collection techniques | ***** data collection |
| **External validity** | *BSC is being implemented in more than one clinical unit (multiple cases) thus increasing the external validity and allowing more room for analytical generalizations.  * Contextualizing to theoretical preposition (Pettigrew’s Framework) | *research design |
| **Reliability** | *Triangulation of methods to give multiple perceptions which can clarify meaning, and verify the repeatability of observations  * Taking detailed notes in a research diary while observing the BSC meetings. This diary also documents our general reflections on study methodology and issues which should be followed up. | *****data collection |

* Tests derived from Yin RK. 2003. *Case Study Research: design and methods,* Sage Publications Inc:
